# A new and updated resource for codon usage tables

**DOI:** 10.1186/s12859-017-1793-7

**Published:** 2017-09-02

**Authors:** John Athey, Aikaterini Alexaki, Ekaterina Osipova, Alexandre Rostovtsev, Luis V. Santana-Quintero, Upendra Katneni, Vahan Simonyan, Chava Kimchi-Sarfaty

**Affiliations:** 1 Division of Plasma Protein Therapeutics, Office of Tissue and Advanced Therapies, Center for Biologics Evaluation and Research, Food and Drug Administration, Silver Spring, USA; 2 High Performance Integrated Environment, Center for Biologics Evaluation and Research, Food and Drug Administration, Silver Spring, USA

**Keywords:** Codon usage bias, Codon optimization, Recombinant protein therapeutics, Translational kinetics

## Abstract

**Background:**

Due to the degeneracy of the genetic code, most amino acids can be encoded by multiple synonymous codons. Synonymous codons naturally occur with different frequencies in different organisms. The choice of codons may affect protein expression, structure, and function. Recombinant gene technologies commonly take advantage of the former effect by implementing a technique termed codon optimization, in which codons are replaced with synonymous ones in order to increase protein expression. This technique relies on the accurate knowledge of codon usage frequencies. Accurately quantifying codon usage bias for different organisms is useful not only for codon optimization, but also for evolutionary and translation studies: phylogenetic relations of organisms, and host-pathogen co-evolution relationships, may be explored through their codon usage similarities. Furthermore, codon usage has been shown to affect protein structure and function through interfering with translation kinetics, and cotranslational protein folding.

**Results:**

Despite the obvious need for accurate codon usage tables, currently available resources are either limited in scope, encompassing only organisms from specific domains of life, or greatly outdated. Taking advantage of the exponential growth of GenBank and the creation of NCBI’s RefSeq database, we have developed a new database, the High-performance Integrated Virtual Environment-Codon Usage Tables (HIVE-CUTs), to present and analyse codon usage tables for every organism with publicly available sequencing data. Compared to existing databases, this new database is more comprehensive, addresses concerns that limited the accuracy of earlier databases, and provides several new functionalities, such as the ability to view and compare codon usage between individual organisms and across taxonomical clades, through graphical representation or through commonly used indices. In addition, it is being routinely updated to keep up with the continuous flow of new data in GenBank and RefSeq.

**Conclusion:**

Given the impact of codon usage bias on recombinant gene technologies, this database will facilitate effective development and review of recombinant drug products and will be instrumental in a wide area of biological research. The database is available at hive.biochemistry.gwu.edu/review/codon.

**Electronic supplementary material:**

The online version of this article (doi:10.1186/s12859-017-1793-7) contains supplementary material, which is available to authorized users.

## Background

There are 64 possible nucleotide triplet combinations but only 20 amino acids to encode; as a result, most amino acids can be encoded by more than one codon. Codons that are translated to the same amino acid are called synonymous. In each organism there is a preference for certain codons over others; therefore, synonymous codons occur with different frequencies, a phenomenon termed codon usage bias, which is observed across species, albeit with different intensities [1]. Two major hypotheses have been proposed for explaining the existence of codon usage bias. One argues that codon usage bias contributes to the efficiency and accuracy of protein translation and is therefore maintained by selection [2]. The other claims that codon usage bias exists because of the non-randomness in mutational patterns, whereby some codons may be more prone to mutation than others and are therefore found at different frequencies [3]. These hypotheses are not mutually exclusive [4–7]. Relative synonymous codon usage (RSCU), Codon adaptation index (CAI) [8], effective number of codons (ENc) [9] and tRNA adaptation index (tAI) [10] are commonly used metrics, aiming to quantitate codon bias in a gene or a species.

We have generated a database, the HIVE-Codon Usage Tables (HIVE-CUTs), presenting the codon usage statistics for every organism that has available compiled sequencing data. The sequence data collected for this analysis have been derived from the GenBank [11] and RefSeq [12] databases. The codon usage tables are linked to a taxonomy tree to allow comparative analysis of the codon usage frequencies. Knowing the frequency of occurrence of codons within a genome is essential in common biological techniques and in a number of fields of study. Codon optimization, which involves replacing rare codons with frequent ones, requires knowledge of the preferred codons in a given organism. Furthermore, synonymous codon usage patterns can be an essential tool in revealing evolutionary relationships between species as well as host-pathogen coevolution and adaptation of pathogens to specific hosts [13–17]. Interestingly, some viruses appear to take advantage of the codon usage of their host to temporally regulate late expression of their proteins [18]. An area of research that is currently gaining attention pertains to how codon usage may affect protein structure. It has long been assumed, based on Anfinsen’s theorem [19], that since synonymous mutations do not affect the primary structure of a protein, they also should not affect the secondary and tertiary structure. However, recent data have suggested that this assumption is untrue; synonymous codon changes can profoundly affect the translation rate of a protein, which in turn may modulate the folding of the nascent polypeptide chain [20–24]. As the translational kinetics of a protein depend, at least in part, on the frequency of its codons, having access to codon usage information can be valuable in determining effects of synonymous mutations on protein structure. It should be noted that synonymous mutations may have multiple other effects on protein expression and function beyond translational kinetics that may instead be linked to effects on nucleosome structure, transcription factor binding, splicing efficiency, RNA-protein interactions, microRNA binding, and RNA secondary structure [24–28]. These effects of synonymous mutations, although of high importance, are not directly related to codon frequency and therefore will not be further discussed here.

Despite the applicability of codon usage tables to many areas of research, currently available resources provide data that are limited, inaccurate or out of date. Some existing databases contain information on bacterial and archaeal genomes but not on eukarya and viruses [29, 30]. The widely used Kazusa database, on the other hand, includes information on all domains of life but has not been updated since 2007 [31]. However, following the rapid development of high-throughput sequencing over the last few years, the amount of sequence information available has drastically increased. The last update of the Kazusa database (GenBank release 160, June 2007) contained just over 3 million coding sequences (CDSs); in comparison, this new database analyses 35 million CDSs from GenBank and another 255 million from RefSeq. For many organisms in the Kazusa database, the number of CDSs included was too low to be useful; for example, the codon usage table for the western lowland gorilla (*Gorilla gorilla gorilla*) was based on only two coding sequences. These shortcomings are widely recognized by researchers who often generate new codon usage tables for the species they are studying [32, 33]. However, this process, in addition to being labour intensive and requiring computational knowledge, may create substantial variability, as the databases from which the sequences are retrieved change over time and different criteria may be applied in the inclusion of sequences for analysis. The presented HIVE-CUTs database, on the other hand, will be updated every 2 months, corresponding with GenBank releases, and each version of the database will remain available to provide a stable reference.

Furthermore, compared to the Kazusa database the HIVE-CUTs database has the advantage of utilizing both GenBank and RefSeq sequences separately. The incorporation of RefSeq data into the proposed database is a critical development that is necessary to provide researchers the most accurate data available, and warrants the creation and maintenance of this new database. NCBI’s RefSeq database aims to minimize redundancy and provide high quality annotations, and provides a data source that was not included in the older database. For example, in the Kazusa database, the sequence for human coagulation factor IX, a single copy gene, was included 13 times due to the inclusion of multiple submissions by various groups. However, the same analysis performed on the RefSeq *Homo sapiens* assembly would include this gene only twice—the wild type sequence and an alternative splicing variant. Overall, larger and more accurate sources of sequencing data have made the generation of current codon usage tables a necessity for a wide range of applications.

## Construction and content

### Input data

Codon usage for all available organisms was computed separately for both the GenBank and RefSeq databases at NCBI. Data from GenBank was derived from GenBank release 215.0 (released August 15 2016 [11]), while RefSeq data [12] used all assemblies that were current as of September 29 2016. Within the RefSeq division, all genome assemblies that were designated “latest” are included in the database. These assemblies were selected by parsing the RefSeq “assembly summary” files available on NCBI. For GenBank, the divisions incorporated into our codon usage database are BCT (bacterial), PRI (primate), ROD (rodent), MAM (other mammalian), VRT (other vertebrate), INV (invertebrate), PLN (plant and fungal), VRL (viral), and PHG (phage). The other divisions published by GenBank do not derive from organismal sources (e.g. the “EST” (expressed sequence tag) division), or come from organisms with no assigned names (e.g. “ENV” (environmental samples)). In total, 288 million coding sequences (35 million from GenBank, 253 million from RefSeq) were included in the database, resulting in the creation of over 855,000 codon usage tables.

### Data processing

The data from both divisions was processed using Python 2.7, using the Biopython module (version 1.68) to parse the annotated genome features [34]. Each record was processed according to the tags available in the file; only protein coding sequences (“CDS” tag) were included for codon usage and GC percent analysis. Pseudogenes and “low quality” proteins (transcripts with a corrected base relative to the genome) were excluded from the analysis. The taxID number of the organism is parsed from the “db_xref” tag in the file, while the scientific name of the organism is retrieved from NCBI’s taxonomy database [35]. Features for which the annotated sequence could not be extracted were also excluded; as a result, records with unusual tags may not have been included, and records that specified their sequence data via another accession record were not included. However, the number of records excluded is low and should not affect the quality of the data overall. Features containing ambiguous nucleotides were included, but the individual codons containing ambiguous nucleotides were excluded. Other information that is parsed from each record includes the translation table and accession number of each individual CDS. The actual execution of data download and parsing was performed using High-performance Integrated Virtual Environment (HIVE) platform [36]. HIVE was originally created and optimized for loading, parsing, storage, and analysis of extra-large datasets.

### Output and organization

The resulting codon usage tables are organized in dictionaries by assembly accession numbers for RefSeq, or by the species name for GenBank. This means that multiple genome submissions for a given organism are combined in GenBank, but are separate entries in RefSeq. Additionally, mitochondrial, chloroplast, plastid, leucoplast, and chromoplast genes are considered separate from the genomic data, and have their own organellar codon usage tables. However, as plasmids are located in the same area as the genome, draw from the same tRNA pools, and use the same genetic code as the genome, plasmid coding sequences are not separated from the organism’s genomic codon usage table. The resulting codon usage tables can be downloaded as a tabular text file, or searched through using our publicly accessible web interface. To obtain the data that best represents the codon usage of an organism, users should search for a single RefSeq assembly, as this is derived from a single sequence assembly from a single organism. GenBank data is compiled from any number of different submissions of varying completion status, and may therefore be skewed when searching for a single organism (see Additional file 1). However, GenBank contains much more data deriving from many more organisms than RefSeq, so even though it may not provide the most accurate data for an organism’s codon usage, it will be of use for less well studied organisms. Additionally, codon usage tables for each CDS, as opposed to each organism, are also produced by the program; these tables cannot currently be viewed via the web, but can be downloaded and parsed. The files available for download through the website are tabular text files comprised of codon usage tables organized either by species/assembly or coding sequence; each entry contains information about the record (e.g. assembly number and DNA type) and the totals for each codon. Table 1 indicates the magnitude of the database, including the number of tables and species included, as well as the distribution of tables between GenBank and RefSeq and between genomic and other organellar tables. Furthermore, each version of the database will be accessible through a stable identifier, allowing researchers to always reference a consistent version of the database.

**Table 1:** HIVE-CUT database size and statistics

Measure	GenBank	RefSeq	Total
Number of tables	781,595	73,817	855,412
Number of species	665,044	37,904	689,420
Genomic tables	353,423	73,553	426,976
Mitochondrial tables	316,820	220	317,040
All plastid tables	111,352	44	111,396
Total number of sequences	34,885,329	253,803,831	288,689,160

This table contains statistics on the data in the database. While the GenBank division contains a much larger number of tables, the number of sequences in each table on average is much higher in RefSeq. The structure of RefSeq assemblies makes them a better representation of genomic codon usage for an organism when available. The HIVE-CUTs database contains substantially more entries than other codon usage databases

The ENc has been adapted from Wright 1990 [9]. Instead of being calculated on a per gene basis, one ENc value is computed for the entirety of the genomic coding sequences. In addition, stop codons are also considered and included in the calculation as any other amino acid. The ENc is also calculated using each different genetic code, and users can select the one most appropriate for their organism of interest.

### Website interface

HIVE is used to implement the data-storage and visualization web-portal for this project. Its interface follows Data-Driven Documents (DDD) paradigm instead of static HTML pages to tackle the visualization challenges presented by the outputs from large data sets [37]. The separation among content, functionality, and object model is the underlying concept of HIVE’s interface achieved by construction of Document Object Model (DOM) model on the client side. This communication is done asynchronously, using an Ajax (Asynchronous Javascript and XML) web application model.

**Fig. 1:**
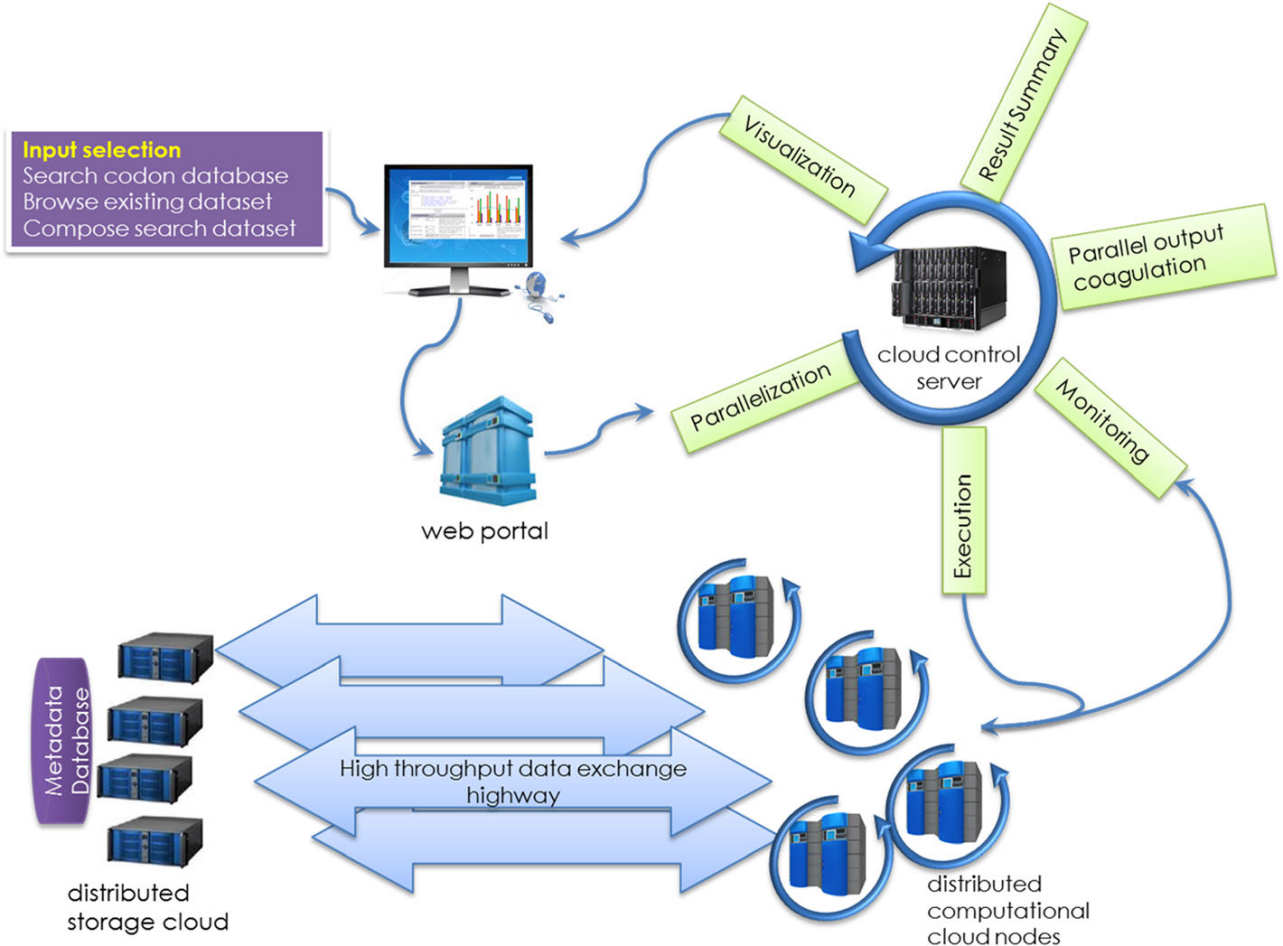
HIVE Platform [36]. A client process submits the information request from the HTML form or web application into the HIVE server; this request is queued for execution and it is computed inside the distributed environment. The front end monitors the status of the request and once the computation is finished, data is retrieved and visualizations are prepared to be sent to the client’s web page

Internally, HIVE is a computer cluster executing a large number of heavily parallelized scientific processes. The front end (user interface) is a simplified representation of an advanced infrastructure that exists on the back end. There are intermediate layers based on Common Gateway Interface (CGI) and SQL database that help with the communication between front end and back end. As the user waits for the service to be completed, a daemon process is responsible for the execution of services, for monitoring progress, and updating the state during the parallel execution. After completion the whole page gets updated with the requested data so the client’s browser can launch specific visualization engines. This allows generation of appropriate interactive visualization. In Fig. 1 there is a general workflow of the HIVE platform.

One of the visualization tools that are used on the page is a dynamic Taxonomy Tree that is constructed using the d3js JavaScript library [37]. The information for this tree is derived from taxonomy data held at NCBI (released May 5 2016 [35]). HIVE’s visualization tree is capable of loading information about a specific node including its taxonomy ID, parent, and children information. The other visualization tool being used is Google Charts that are integrated into HIVE’s visual library. Google Charts are customizable on many parameters, and these parameters are used to generate a bar chart that allows the users to make a side by side comparison of selected codon tables.

Users can access additional webpages and resources that utilize codon usage tables through the “Other Resources” tab on the webpage.

## Utility and discussion

**Fig. 2:**
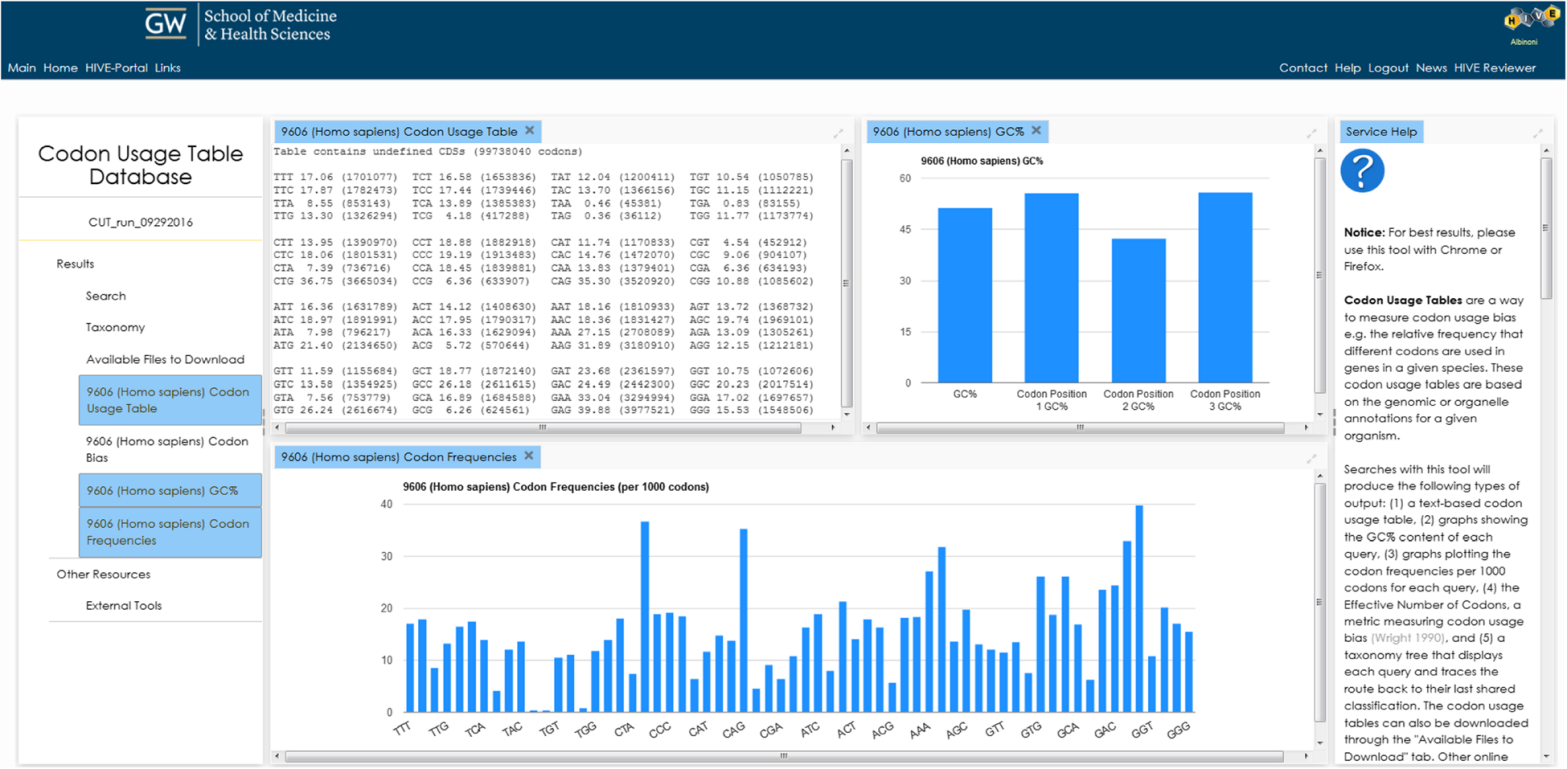
Screenshot of HIVE-CUTs webpage with *Homo sapiens* results. Results include codon usage frequencies per 1000 codons as a plain text table (top left) and graph (bottom), in the default order specified by NCBI’s standard genetic code definition. The GC frequency in the genome and at each codon position is also presented in a graph (top right). The help panel is included (right)

**Fig. 3:**
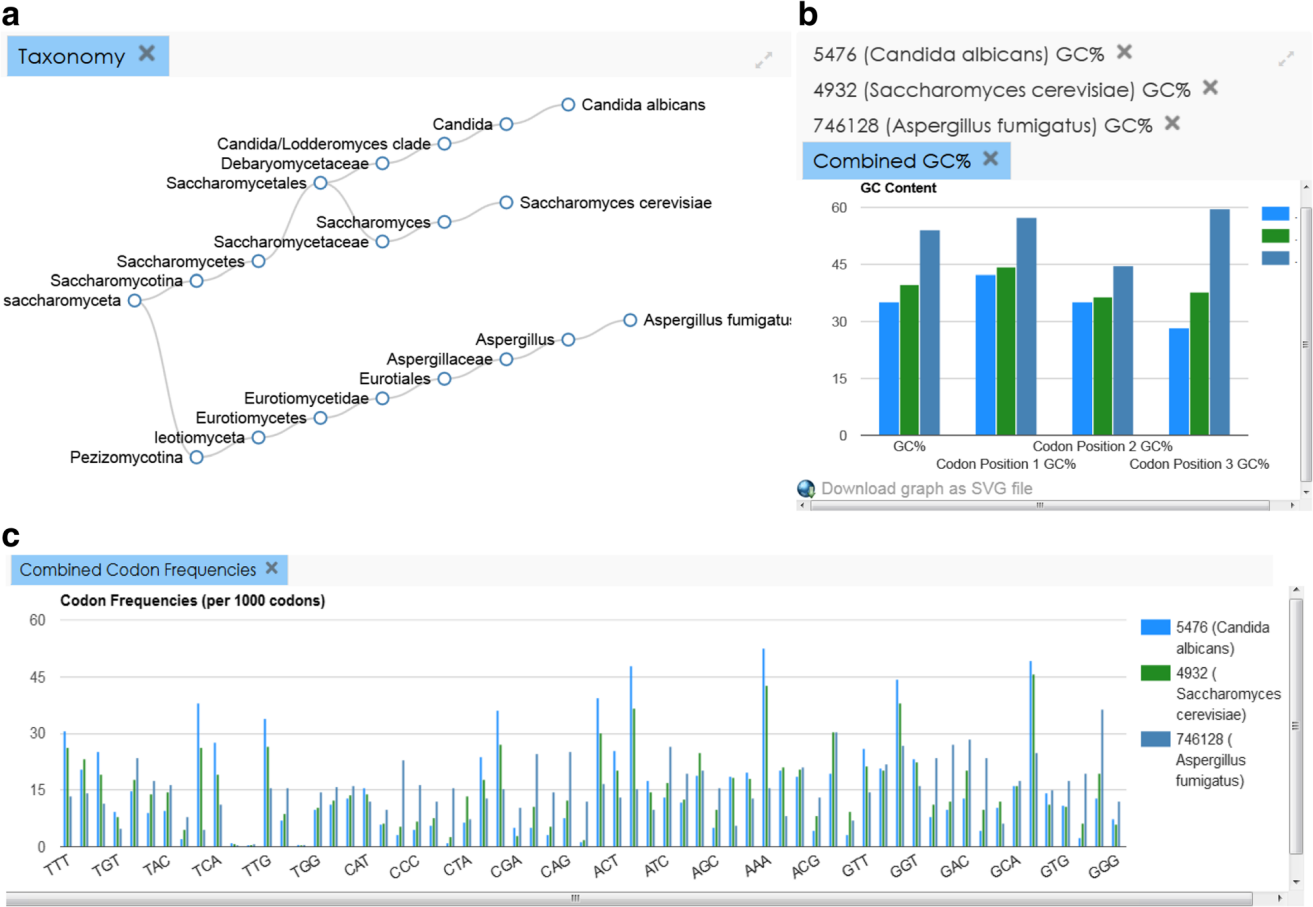
Screenshots of HIVE-CUTs webpage with *Candida albicans*, *Saccharomyces cerevisiae,* and *Aspergillus fumigatus* results. **a** Taxonomy tree showing the evolutionary relationship between the species. **b** The GC frequency in the genome and at each position of the codon plotted for all three species for comparison. **c** Codon frequencies per 1000 codons plotted for all three species

We have generated new codon usage tables for every organism in GenBank and RefSeq and created a user-friendly platform where codon usage data can be retrieved from a publically available website [38]. The initial HIVE-CUTs webpage contains a search tab, results tab, and help tabs on the right and bottom. The web interface for searching the database features several options for searching the data. Users must decide whether to search through GenBank or RefSeq, as well as what type of data (genomic or another organelle) they want to analyse. Users can search for entries based on scientific names, taxonomical ID numbers (taxID), or (in RefSeq only) assembly accessions. All of these options, except searching for a single assembly, can be applied to any taxonomical rank, allowing users to retrieve and compare data from different clades. When searching for a species-rank scientific name or taxID number, users also have the option to combine entries belonging to sub-species of that entry, by choosing the “deep search” option, e.g. retrieving *E. coli* and all its strains, or only retrieving submissions for *E. coli* with no strain information assigned. Once a search is submitted the results appear in several tables and graphs. Each window and graph can be enlarged or closed. The codon usage tables are in plain text format with each codon, its frequency per 1000 codons, and the raw total for that codon in the genome, in the default order specified by NCBI’s standard genetic code definition (Fig. 2). This is a common format and the table can be copied and directly pasted in a number of applications such as ATGme [39] and Rare Codon Calculator: %MinMax [40]; several such tools are linked directly from the database webpage. Each search will produce two graphs, one plotting the GC percent frequency of the organism’s coding sequences as well as at each codon position, and the other plotting the frequencies of each codon per 1000 codons. The graphical presentation of the frequencies of each codon (Fig. 2) can be especially useful when comparing frequencies across different codon usage tables. To enable comparisons between different organisms and clades, multiple queries can be submitted simultaneously; the codon and GC frequencies for each query are plotted both individually and together (Fig. 3). In addition, a text table listing the ENc for each query is generated. ENc is a metric that measures codon bias in terms of deviation from an assumed neutral distribution of synonymous codon usage. Larger ENc values correspond to more equal usage of synonymous codons, while the lowest possible ENc value would result from the case of one codon used for each amino acid [9]. The ENc was calculated for all genomic coding sequences collectively. RefSeq and GenBank do not always assign a genetic code to each genome, therefore, ENc was calculated using each genetic code; users may select the ENc that is appropriate for their organisms of interest. To facilitate studying the evolution of codon usage bias across species we incorporated a visual representation of their taxonomical relationship in our application. When users submit a search for one or more taxonomical nodes, the nodes will be highlighted and the branch will be expanded to show the relationship between them, going back to the highest common classification. Though the tree is taxonomical, not phylogenetic, being able to visualize the distance between organisms of interest and in parallel examine their similarities in terms of codon usage bias can be an instrumental tool in evolutionary studies. In the example shown, *Candida albicans* and *Saccharomyces cerevisiae*, two taxonomically close yeast species, are shown along with *Aspergillus fumingatus,* which is more distantly related. The distance in the taxonomy tree is reflected in the differences of their codon usage tables (Fig. 3).

The HIVE-CUTs may be instrumental in recombinant gene applications such as gene therapy, vaccine development and protein therapeutics, and in a wide area of research including evolution, comparative molecular biology and translation kinetics.

**Fig. 4:**
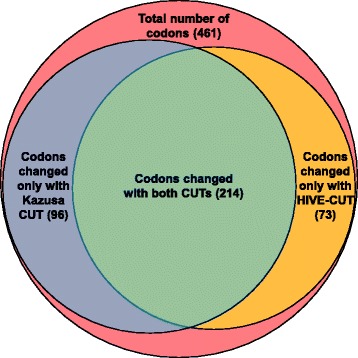
Differences in codon optimization based on the HIVE-CUT and the Kazusa codon usage tables. The HIVE-CUT and the Kazusa codon usage tables were entered in the codon optimization algorithm ATGme to determine the number of suboptimal codons [39]. The Venn diagram shows how many codons were determined to be sub-optimal in the human coagulation factor IX gene for expression in CHO (*Cricetulus griseus)* cells. The codon usage tables used appear in Additional file 2

Knowledge of codon usage across species is crucial when recombinant proteins are expressed in heterologous organisms [41–44]. There are several approaches that are commonly used to increase expression of heterologous proteins, including codon optimization [45–47], codon harmonization [48, 49], and supplementation of rare tRNAs [50–53]. When codon usage in the organism of origin is starkly different from that of the organism used for expression, for example when a human gene is expressed in *E. coli*, codon optimization (i.e. replacement of rare codons with more frequent synonymous codons) can to an increase in expression of a few orders of magnitude [41, 45, 54, 55]. Codon harmonization is conceptually similar to codon optimization, wherein codons of the native protein are replaced with synonymous codons that have a similar usage frequency in the heterologous expression host [48, 49]. However, accurate codon usage data is important for ensuring that optimization or harmonization strategies actually lead to improved expression; if the codon usage data is incomplete or inaccurate, optimization steps could be unsuccessful at increasing expression or may even reduce it. For example, the biotechnology industry often uses Chinese hamster ovary (CHO) cells to express human recombinant proteins. *Homo sapiens* and *Cricetulus griseus* (Chinese hamster) are not very different in terms of their codon usage bias. Furthermore, in 2007, *Cricetulus griseus* had not been extensively sequenced, and therefore its codon usage tables were not accurate. Using human coagulation factor IX, a gene that is commonly codon optimized for clinical applications like gene therapy [56–58], as an example, it is clear that accurate codon usage data is critical for optimization strategies. As illustrated in Fig. 4, when using codon usage tables that were last updated in 2007 to optimize the human coagulation factor IX gene for expression in CHO cells using ATGme [39], 310 codons are identified as suboptimal. Performing the same analysis with HIVE-CUTs resulted in 287 optimized codons, only 214 of which were the same as when using the older tables. Performing codon optimization or harmonization based on inaccurate codon usage tables would, therefore, be ineffective. Alternatively, when the tRNA supplementation approach is used, codon usage, of a single gene or the entire genome, can be compared to tRNA levels to determine which tRNA may require supplementation. Availability of tRNA can be either estimated computationally [10, 59] or measured experimentally.

An area of research that has been recently gaining attention is whether synonymous codon substitutions have effects on protein translation beyond levels of expression [32, 60–62]. Codon frequency has coevolved to correlate with tRNA concentration [63–66]. As a result, translational efficiency at the codon level is affected by tRNA abundance. It is generally accepted that rare codons are translated slower than the common ones and it has been shown that rare codons often cluster [40]. These rare codon clusters can induce pauses during translation that have experimentally been shown to affect cotranslational folding [20]. Although there is little consensus regarding patterns of rare codon clusters and secondary protein structure, there is data from an array of yeast species suggesting that coil regions of a protein are depleted of common codons while β-sheets are depleted of rare codons [67]. Interestingly, coil regions are comprised of loops that fold before exit of the ribosomal tunnel. In contrast, β-sheet domains are topologically discontinuous and must await synthesis to begin folding [67]. Collectively this data supports a causal relationship between codon choice, translation rate and protein structure.

**Fig. 5:**
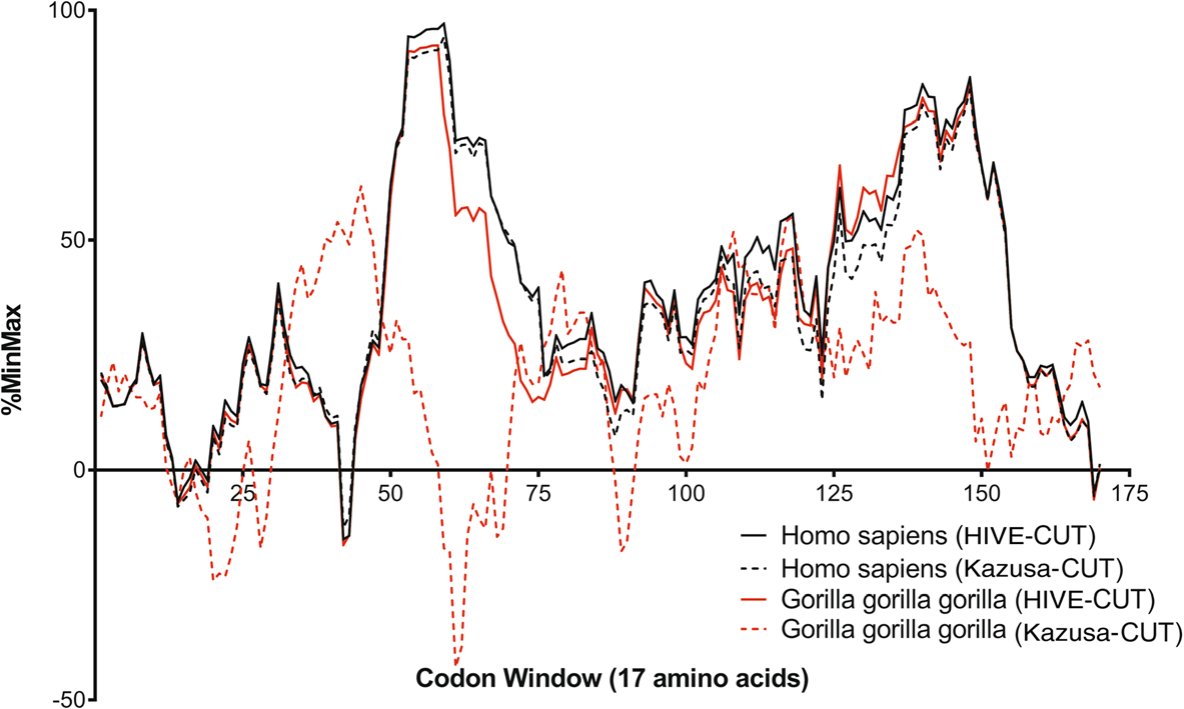
Rare codon cluster distribution based on the HIVE-CUT and the Kazusa codon usage tables. The %MinMax algorithm [40] was implemented to generate results for the interferon beta-1b gene sequence of *Homo sapiens* and *Gorilla gorilla gorilla*. The human and gorilla proteins have similar amino acid sequences and show similar results with the HIVE-CUT; however, highly divergent results were observed with Kazusa CUTs. The codon usage tables for these species used in the calculation of the translation rate appear in Additional file 3

The availability of codon usage tables that span a very wide range of species can be instrumental in unravelling the role of codon choice on co-translational folding. Algorithms that evaluate the relative rareness of codons in a nucleotide sequence used to produce a given protein sequence [40] can serve as a rough proxy for the local translation rate, and the presence of translational pauses due to rare codons can be studied. To obtain a more accurate estimation of rare codon clusters, accurate codon usage tables are required. For example, when examining the rare codon distribution of the human interferon beta-1b a number of potential translational pauses are apparent. A similar pattern of rare codon clusters is also observed with the gorilla sequence of interferon beta-1b, which is due both to the similarity of the amino acid sequence in the two species but also due to similarities in codon usage bias (Fig. 5). If, however, an older codon usage table had been used, a dramatically different pattern of rare codon clusters would have been generated giving rise to false conclusions (Fig. 5). Comparing the rare codon distribution of a human protein to those of closely related species has proven useful in determining the functional role of synonymous mutations [67] and how they may cause disease [32].

## Conclusions

Codon usage bias plays a role in many biological processes, and substitution of synonymous codons is a very common technique in industry and research. Accurate codon usage data is an important part of many common bioinformatics tools that incorporate the effects of codon usage bias into their analyses. This database is a dramatic improvement over existing databases. It is more comprehensive in terms of the number of species included and more accurate due to the vastly larger sources and improved quality of sequencing data and their associated annotations.

## Availability and requirements

**Project name:** HIVE-Codon Usage Tables

**Project home page:** https://hive.biochemistry.gwu.edu/review/codon

**Operating system:** Platform independent

**Programming languages:** Python 2.7, Javascript, C++

**Other requirements:** Web browser (Chrome or Firefox)

**License:** The database is publicly available

## Additional file

Additional file 1: HIVE-CUT screenshot showing search results for *Homo sapiens* using the RefSeq and GenBank databases. (DOCX 154 kb)
Additional file 2:
*Homo sapiens* and *Cricetulus griseus* CUTs. This table contains the data used to create Fig. 4 of the main text. (DOCX 22 kb)
Additional file 3:
*Homo sapiens* and *Gorilla gorilla gorilla* CUTs. This table contains the data used to create Fig. 5 of the main text. (DOCX 24 kb)

## Abbreviations

BCT: Bacterial (GenBank division)
CDS: Coding DNA sequence
CGI: Common gateway interface
CHO: Chinese hamster ovary
CUT: Codon usage table
DDD: Data-driven documents
DNA: Deoxyribonucleic acid
DOM: Document object model
ENV: Environmental (GenBank division)
EST: Expressed sequence tag (GenBank division)
GC: Guanine-cytosine
HIVE: High-performance integrated virtual environment
HTML: Hypertext markup language
INV: invertebrate (GenBank division)
MAM: Other mammalian (GenBank division)
NCBI: National center for biotechnology information
PHG: Phage (GenBank division)
PLN: Plant and fungal (GenBank division)
PRI: Primate (GenBank division)
RNA: Ribonucleic acid
ROD: Rodent (GenBank division)
SQL: Structured query language
taxID: Taxonomy ID number
tRNA: Transfer RNA
VRL: Viral (GenBank division)
VRT: Other vertebrate (GenBank division)
XML: Extensible markup language
