# Supplementary material for: A new and updated resource for codon usage tables
Source: BMC Bioinformatics. 2017 Sep 2;18:391. doi: 10.1186/s12859-017-1793-7 (PMC5581930; doi:10.1186/s12859-017-1793-7)

Additional file 1. HIVE-CUT screenshot showing search results for *Homo sapiens* using the RefSeq and GenBank databases.


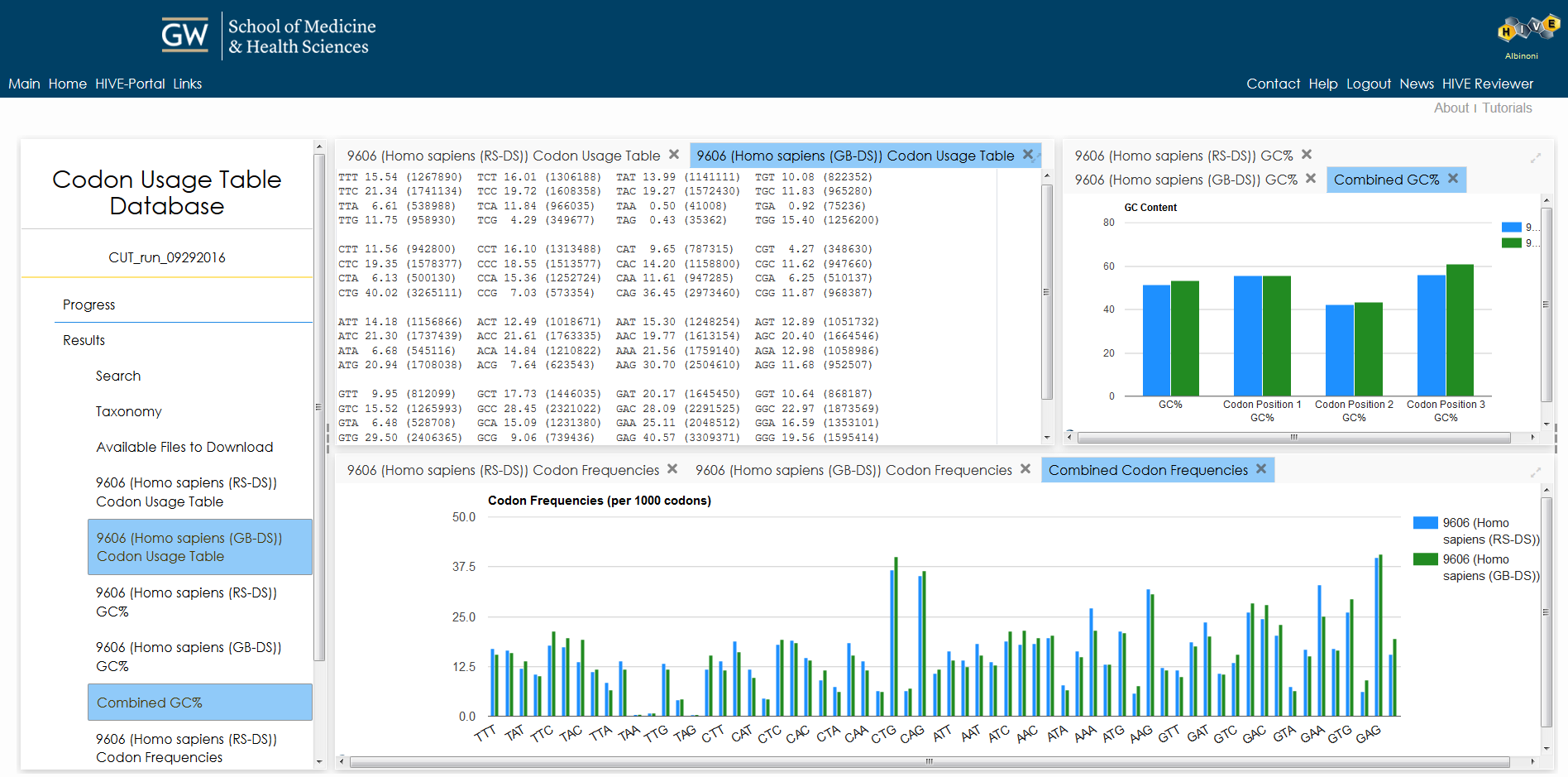

Supplement: Additional file 1: — HIVE-CUT screenshot showing search results for Homo sapiens using the RefSeq and GenBank databases. (DOCX 154 kb) [file 12859_2017_1793_MOESM1_ESM.docx]
