# Supplementary material for: A new and updated resource for codon usage tables
Source: BMC Bioinformatics. 2017 Sep 2;18:391. doi: 10.1186/s12859-017-1793-7 (PMC5581930; doi:10.1186/s12859-017-1793-7)
Supplement: Additional file 2: — Homo sapiens and Cricetulus griseus CUTs. This table contains the data used to create Fig. 4 of the main text. (DOCX 22 kb) [file 12859_2017_1793_MOESM2_ESM.docx]

Additional file 2: *Homo sapiens* and *Cricetulus griseus* CUTs.

A) *Homo sapiens* – HIVE-CUT

| TTT 17.06 (1701077) | TTC 17.87 (1782473) | TTA 8.55 (853143) | TTG 13.30 (1326294) |
| --- | --- | --- | --- |
| CTT 13.95 (1390970) | CTC 18.06 (1801531) | CTA 7.39 (736716) | CTG 36.75 (3665034) |
| ATT 16.36 (1631789) | ATC 18.97 (1891991) | ATA 7.98 (796217) | ATG 21.40 (2134650) |
| GTT 11.59 (1155684) | GTC 13.58 (1354925) | GTA 7.56 (753779) | GTG 26.24 (2616674) |
|  |  |  |  |
| TAT 12.04 (1200411) | TAC 13.70 (1366156) | TAA 0.46 (45381) | TAG 0.36 (36112) |
| CAT 11.74 (1170833) | CAC 14.76 (1472070) | CAA 13.83 (1379401) | CAG 35.30 (3520920) |
| AAT 18.16 (1810933) | AAC 18.36 (1831427) | AAA 27.15 (2708089) | AAG 31.89 (3180910) |
| GAT 23.68 (2361597) | GAC 24.49 (2442300) | GAA 33.04 (3294994) | GAG 39.88 (3977521) |
|  |  |  |  |
| TCT 16.58 (1653836) | TCC 17.44 (1739446) | TCA 13.89 (1385383) | TCG 4.18 (417288) |
| CCT 18.88 (1882918) | CCC 19.19 (1913483) | CCA 18.45 (1839881) | CCG 6.36 (633907) |
| ACT 14.12 (1408630) | ACC 17.95 (1790317) | ACA 16.33 (1629094) | ACG 5.72 (570644) |
| GCT 18.77 (1872140) | GCC 26.18 (2611615) | GCA 16.89 (1684588) | GCG 6.26 (624561) |
|  |  |  |  |
| TGT 10.54 (1050785) | TGC 11.15 (1112221) | TGA 0.83 (83155) | TGG 11.77 (1173774) |
| CGT 4.54 (452912) | CGC 9.06 (904107) | CGA 6.36 (634193) | CGG 10.88 (1085602) |
| AGT 13.72 (1368732) | AGC 19.74 (1969101) | AGA 13.09 (1305261) | AGG 12.15 (1212181) |
| GGT 10.75 (1072606) | GGC 20.23 (2017514) | GGA 17.02 (1697657) | GGG 15.53 (1548506) |

B) *Homo sapiens* - Kazusa CUT

| TTT 17.6 (714298) | TTC 20.3 (824692) | TTA 7.7 (311881) | TTG 12.9 (525688) |
| --- | --- | --- | --- |
| CTT 13.2 (536515) | CTC 19.6 (796638) | CTA 7.2 (290751) | CTG 39.6 (1611801) |
| ATT 16.0 (650473) | ATC 20.8 (846466) | ATA 7.5 (304565) | ATG 22.0 (896005) |
| GTT 11.0 (448607) | GTC 14.5 (588138) | GTA 7.1 (287712) | GTG 28.1 (1143534) |
|  |  |  |  |
| TAT 12.2 (495699) | TAC 15.3 (622407) | TAA 1.0 (40285) | TAG 0.8 ( 32109) |
| CAT 10.9 (441711) | CAC 15.1 (613713) | CAA 12.3 (501911) | CAG 34.2 (1391973) |
| AAT 17.0 (689701) | AAC 19.1 (776603) | AAA 24.4 (993621) | AAG 31.9 (1295568) |
| GAT 21.8 (885429) | GAC 25.1 (1020595) | GAA 29.0 (1177632) | GAG 39.6 (1609975) |
|  |  |  |  |
| TCT 15.2 (618711) | TCC 17.7 (718892) | TCA 12.2 (496448) | TCG 4.4 (179419) |
| CCT 17.5 (713233) | CCC 19.8 (804620) | CCA 16.9 (688038) | CCG 6.9 (281570) |
| ACT 13.1 (533609) | ACC 18.9 (768147) | ACA 15.1 (614523) | ACG 6.1 (246105) |
| GCT 18.4 (750096) | GCC 27.7 (1127679) | GCA 15.8 (643471) | GCG 7.4 (299495) |
|  |  |  |  |
| TGT 10.6 (430311) | TGC 12.6 (513028) | TGA 1.6 ( 63237) | TGG 13.2 (535595) |
| CGT 4.5 (184609) | CGC 10.4 (423516) | CGA 6.2 (250760) | CGG 11.4 (464485) |
| AGT 12.1 (493429) | AGC 19.5 (791383) | AGA 12.2 (494682) | AGG 12.0 (486463) |
| GGT 10.8 (437126) | GGC 22.2 (903565) | GGA 16.5 (669873) | GGG 16.5 (669768) |

C) *Cricetulus griseus* – HIVE-CUT

| TTT 17.27 (688965) | TCT 17.81 (710575) | TAT 12.18 (486071) | TGT 11.76 (469155) |
| --- | --- | --- | --- |
| TTC 18.88 (753245) | TCC 17.51 (698606) | TAC 13.86 (552771) | TGC 11.10 (442910) |
| TTA 7.77 (310131) | TCA 14.25 (568386) | TAA 0.74 (29676) | TGA 1.59 (63570) |
| TTG 14.36 (572691) | TCG 3.37 (134420) | TAG 0.63 (25159) | TGG 12.16 (484893) |
|  |  |  |  |
| CTT 14.71 (586847) | CCT 20.10 (801829) | CAT 12.56 (501049) | CGT 4.83 (192543) |
| CTC 18.24 (727802) | CCC 17.24 (687740) | CAC 14.65 (584527) | CGC 7.51 (299583) |
| CTA 8.58 (342158) | CCA 19.46 (776269) | CAA 14.03 (559605) | CGA 6.99 (278769) |
| CTG 35.98 (1435506) | CCG 4.44 (177183) | CAG 35.34 (1409706) | CGG 9.36 (373217) |
|  |  |  |  |
| ATT 16.05 (640173) | ACT 15.00 (598191) | AAT 17.23 (687400) | AGT 14.75 (588411) |
| ATC 19.59 (781410) | ACC 17.32 (691022) | AAC 18.77 (748716) | AGC 19.28 (769156) |
| ATA 7.75 (309290) | ACA 17.77 (708770) | AAA 25.34 (1011034) | AGA 13.75 (548490) |
| ATG 22.12 (882572) | ACG 4.27 (170439) | AAG 33.32 (1329268) | AGG 12.85 (512493) |
|  |  |  |  |
| GTT 11.63 (464065) | GCT 20.59 (821354) | GAT 23.31 (929848) | GGT 11.72 (467410) |
| GTC 13.97 (557137) | GCC 23.56 (939971) | GAC 24.50 (977553) | GGC 18.48 (737156) |
| GTA 8.05 (321115) | GCA 17.51 (698414) | GAA 31.62 (1261453) | GGA 17.34 (691556) |
| GTG 26.17 (1044036) | GCG 4.10 (163572) | GAG 38.55 (1537975) | GGG 14.46 (577024) |

D) *Cricetulus griseus* – Kazusa CUT

| TTT 19.6 (3005) | TCT 16.0 (2450) | TAT 13.1 (2017) | TGT 9.1 (1397) |
| --- | --- | --- | --- |
| TTC 22.0 (3381) | TCC 16.5 (2529) | TAC 16.4 (2519) | TGC 10.3 (1589) |
| TTA 6.4 (978) | TCA 10.3 (1577) | TAA 0.6 (93) | TGA 1.2 (177) |
| TTG 14.1 (2169) | TCG 3.4 (529) | TAG 0.5 (84) | TGG 13.1 (2012) |
|  |  |  |  |
| CTT 13.2 (2023) | CCT 16.7 (2563) | CAT 10.2 (1563) | CGT 5.6 (863) |
| CTC 18.4 (2818) | CCC 17.0 (2608) | CAC 12.9 (1980) | CGC 9.3 (1429) |
| CTA 7.6 (1174) | CCA 15.6 (2388) | CAA 10.3 (1587) | CGA 7.2 (1102) |
| CTG 38.8 (5955) | CCG 4.3 (657) | CAG 33.4 (5122) | CGG 10.1 (1558) |
|  |  |  |  |
| ATT 17.4 (2673) | ACT 14.1 (2172) | AAT 17.4 (2671) | AGT 11.4 (1756) |
| ATC 24.8 (3808) | ACC 20.3 (3118) | AAC 21.2 (3248) | AGC 16.4 (2521) |
| ATA 6.9 (1053) | ACA 15.7 (2418) | AAA 24.6 (3782) | AGA 10.1 (1557) |
| ATG 23.0 (3538) | ACG 4.5 (685) | AAG 38.4 (5895) | AGG 10.2 (1570) |
|  |  |  |  |
| GTT 11.6 (1780) | GCT 22.4 (3432) | GAT 24.6 (3781) | GGT 12.8 (1968) |
| GTC 15.7 (2408) | GCC 25.9 (3973) | GAC 28.1 (4310) | GGC 21.3 (3268) |
| GTA 7.8 (1202) | GCA 16.3 (2497) | GAA 28.4 (4355) | GGA 15.8 (2425) |
| GTG 30.1 (4628) | GCG 5.0 (765) | GAG 41.1 (6311) | GGG 13.4 (2063) |
