# Supplementary material for: A new and updated resource for codon usage tables
Source: BMC Bioinformatics. 2017 Sep 2;18:391. doi: 10.1186/s12859-017-1793-7 (PMC5581930; doi:10.1186/s12859-017-1793-7)
Supplement: Additional file 3: — Homo sapiens and Gorilla gorilla gorilla CUTs. This table contains the data used to create Fig. 5 of the main text. (DOCX 24 kb) [file 12859_2017_1793_MOESM3_ESM.docx]

Additional file 3: *Homo sapiens* and *Gorilla gorilla gorilla* CUTs.

1. *Homo sapiens* – HIVE-CUT

| TTT 17.06 (1701077) | TTC 17.87 (1782473) | TTA 8.55 (853143) | TTG 13.30 (1326294) |
| --- | --- | --- | --- |
| CTT 13.95 (1390970) | CTC 18.06 (1801531) | CTA 7.39 (736716) | CTG 36.75 (3665034) |
| ATT 16.36 (1631789) | ATC 18.97 (1891991) | ATA 7.98 (796217) | ATG 21.40 (2134650) |
| GTT 11.59 (1155684) | GTC 13.58 (1354925) | GTA 7.56 (753779) | GTG 26.24 (2616674) |
|  |  |  |  |
| TAT 12.04 (1200411) | TAC 13.70 (1366156) | TAA 0.46 (45381) | TAG 0.36 (36112) |
| CAT 11.74 (1170833) | CAC 14.76 (1472070) | CAA 13.83 (1379401) | CAG 35.30 (3520920) |
| AAT 18.16 (1810933) | AAC 18.36 (1831427) | AAA 27.15 (2708089) | AAG 31.89 (3180910) |
| GAT 23.68 (2361597) | GAC 24.49 (2442300) | GAA 33.04 (3294994) | GAG 39.88 (3977521) |
|  |  |  |  |
| TCT 16.58 (1653836) | TCC 17.44 (1739446) | TCA 13.89 (1385383) | TCG 4.18 (417288) |
| CCT 18.88 (1882918) | CCC 19.19 (1913483) | CCA 18.45 (1839881) | CCG 6.36 (633907) |
| ACT 14.12 (1408630) | ACC 17.95 (1790317) | ACA 16.33 (1629094) | ACG 5.72 (570644) |
| GCT 18.77 (1872140) | GCC 26.18 (2611615) | GCA 16.89 (1684588) | GCG 6.26 (624561) |
|  |  |  |  |
| TGT 10.54 (1050785) | TGC 11.15 (1112221) | TGA 0.83 (83155) | TGG 11.77 (1173774) |
| CGT 4.54 (452912) | CGC 9.06 (904107) | CGA 6.36 (634193) | CGG 10.88 (1085602) |
| AGT 13.72 (1368732) | AGC 19.74 (1969101) | AGA 13.09 (1305261) | AGG 12.15 (1212181) |
| GGT 10.75 (1072606) | GGC 20.23 (2017514) | GGA 17.02 (1697657) | GGG 15.53 (1548506) |

1. *Homo sapiens* - Kazusa CUT

| TTT 17.6 (714298) | TTC 20.3 (824692) | TTA 7.7 (311881) | TTG 12.9 (525688) |
| --- | --- | --- | --- |
| CTT 13.2 (536515) | CTC 19.6 (796638) | CTA 7.2 (290751) | CTG 39.6 (1611801) |
| ATT 16.0 (650473) | ATC 20.8 (846466) | ATA 7.5 (304565) | ATG 22.0 (896005) |
| GTT 11.0 (448607) | GTC 14.5 (588138) | GTA 7.1 (287712) | GTG 28.1 (1143534) |
|  |  |  |  |
| TAT 12.2 (495699) | TAC 15.3 (622407) | TAA 1.0 (40285) | TAG 0.8 ( 32109) |
| CAT 10.9 (441711) | CAC 15.1 (613713) | CAA 12.3 (501911) | CAG 34.2 (1391973) |
| AAT 17.0 (689701) | AAC 19.1 (776603) | AAA 24.4 (993621) | AAG 31.9 (1295568) |
| GAT 21.8 (885429) | GAC 25.1 (1020595) | GAA 29.0 (1177632) | GAG 39.6 (1609975) |
|  |  |  |  |
| TCT 15.2 (618711) | TCC 17.7 (718892) | TCA 12.2 (496448) | TCG 4.4 (179419) |
| CCT 17.5 (713233) | CCC 19.8 (804620) | CCA 16.9 (688038) | CCG 6.9 (281570) |
| ACT 13.1 (533609) | ACC 18.9 (768147) | ACA 15.1 (614523) | ACG 6.1 (246105) |
| GCT 18.4 (750096) | GCC 27.7 (1127679) | GCA 15.8 (643471) | GCG 7.4 (299495) |
|  |  |  |  |
| TGT 10.6 (430311) | TGC 12.6 (513028) | TGA 1.6 ( 63237) | TGG 13.2 (535595) |
| CGT 4.5 (184609) | CGC 10.4 (423516) | CGA 6.2 (250760) | CGG 11.4 (464485) |
| AGT 12.1 (493429) | AGC 19.5 (791383) | AGA 12.2 (494682) | AGG 12.0 (486463) |
| GGT 10.8 (437126) | GGC 22.2 (903565) | GGA 16.5 (669873) | GGG 16.5 (669768) |
|  |  |  |  |

1. *Gorilla gorilla gorilla* – HIVE-CUT

| TTT 17.13 (279830) | TTC 19.39 (316681) | TTA 7.73 (126278) | TTG 12.90 (210769) |
| --- | --- | --- | --- |
| CTT 13.45 (219747) | CTC 19.03 (310814) | CTA 7.16 (116907) | CTG 38.57 (629990) |
| ATT 15.79 (257928) | ATC 19.90 (325006) | ATA 7.38 (120546) | ATG 21.55 (352068) |
| GTT 11.01 (179909) | GTC 14.10 (230374) | GTA 7.15 (116758) | GTG 27.25 (445131) |
|  |  |  |  |
| TAT 11.84 (193364) | TAC 14.32 (233990) | TAA 0.71 (11668) | TAG 0.54 (8886) |
| CAT 11.20 (182920) | CAC 15.15 (247421) | CAA 12.66 (206796) | CAG 34.50 (563542) |
| AAT 17.05 (278516) | AAC 18.60 (303911) | AAA 25.11 (410185) | AAG 32.05 (523546) |
| GAT 22.14 (361687) | GAC 24.68 (403213) | GAA 30.05 (490854) | GAG 39.76 (649563) |
|  |  |  |  |
| TCT 15.49 (253109) | TCC 17.65 (288317) | TCA 12.73 (207989) | TCG 4.44 (72514) |
| CCT 18.40 (300560) | CCC 20.27 (331121) | CCA 17.78 (290463) | CCG 7.07 (115451) |
| ACT 13.39 (218759) | ACC 18.30 (298894) | ACA 15.20 (248277) | ACG 5.93 (96798) |
| GCT 18.75 (306305) | GCC 27.60 (450848) | GCA 16.38 (267656) | GCG 7.19 (117514) |
|  |  |  |  |
| TGT 10.70 (174826) | TGC 12.26 (200244) | TGA 1.33 (21670) | TGG 12.57 (205256) |
| CGT 4.66 (76144) | CGC 10.37 (169320) | CGA 6.31 (103110) | CGG 11.68 (190744) |
| AGT 12.70 (207458) | AGC 19.77 (322965) | AGA 12.43 (203082) | AGG 12.34 (201615) |
| GGT 10.92 (178340) | GGC 21.96 (358796) | GGA 16.93 (276487) | GGG 16.65 (272063) |

1. *Gorilla gorilla gorilla* - Kazusa CUT

| TTT 46.7 (28) | TTC 30.1 (18) | TTA 13.4 (8) | TTG 11.7 (7) |
| --- | --- | --- | --- |
| CTT 30.1 (18) | CTC 21.7 (13) | CTA 6.7 (4) | CTG 31.7 (19) |
| ATT 21.7 (13) | ATC 41.7 (25) | ATA 11.7 (7) | ATG 20.0 (12) |
| GTT 13.4 (8) | GTC 18.4 (11) | GTA 8.3 (5) | GTG 26.7 (16) |
|  |  |  |  |
| TAT 23.4 (14) | TAC 20.0 (12) | TAA 1.7 (1) | TAG 0.0 (0) |
| CAT 15.0 (9) | CAC 8.3 (5) | CAA 21.7 (13) | CAG 20.0 (12) |
| AAT 16.7 (10) | AAC 18.4 (11) | AAA 28.4 (17) | AAG 31.7 (19) |
| GAT 20.0 (12) | GAC 16.7 (10) | GAA 31.7 (19) | GAG 20.0 (12) |
|  |  |  |  |
| TCT 18.4 (11) | TCC 3.3 (2) | TCA 11.7 (7) | TCG 5.0 (3) |
| CCT 5.0 (3) | CCC 10.0 (6) | CCA 18.4 (11) | CCG 1.7 (1) |
| ACT 20.0 (12) | ACC 15.0 (9) | ACA 18.4 (11) | ACG 6.7 (4) |
| GCT 21.7 (13) | GCC 13.4 (8) | GCA 8.3 (5) | GCG 3.3 (2) |
|  |  |  |  |
| TGT 11.7 (7) | TGC 21.7 (13) | TGA 1.7 (1) | TGG 16.7 (10) |
| CGT 1.7 (1) | CGC 5.0 (3) | CGA 6.7 (4) | CGG 3.3 (2) |
| AGT 16.7 (10) | AGC 6.7 (4) | AGA 6.7 (4) | AGG 16.7 (10) |
| GGT 11.7 (7) | GGC 11.7 (7) | GGA 20.0 (12) | GGG 13.4 (8) |
